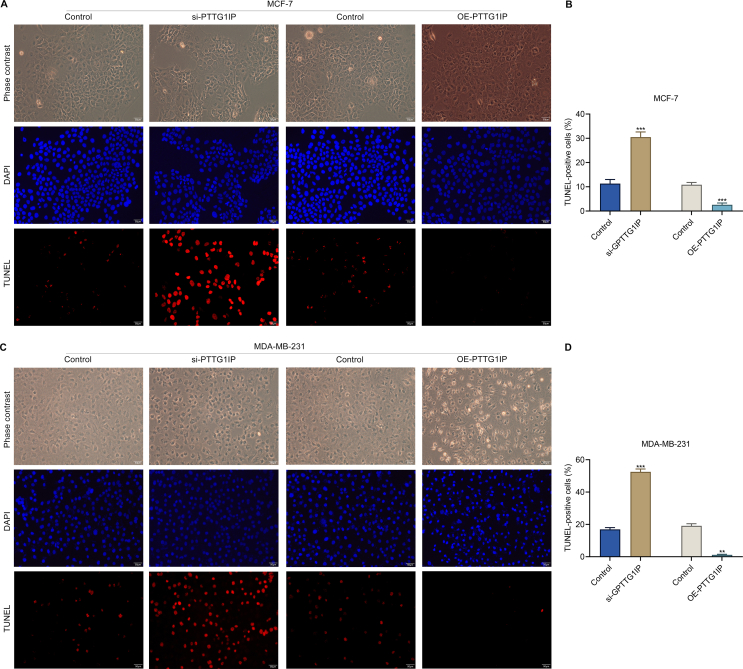# Disulfidptosis heterogeneity in breast cancer uncovers PTTG1IP as an actionable therapeutic target

**DOI:** 10.1016/j.gendis.2024.101257

**Published:** 2024-03-05

**Authors:** Kun Fang, Suxiao Jiang, Zhengjie Xu, Meng Luo, Changsheng Yan

**Affiliations:** a Department of Surgery, Yinchuan Maternal and Child Health Hospital, Yinchuan, Ningxia 750001, China; b Department of Surgery, The First Affiliated Hospital of Harbin Medical University, Harbin, Heilongjiang 150001, China

Breast cancer (BRCA) is the dominating form of cancer affecting women, with an estimated 2.3 million newly diagnosed cases and 685,000 deaths in 2020.^1^ An emerging study has proposed a novel metabolism-related regulated cell death modality called disulfidptosis induced by abnormal accumulation of intracellular disulfides in SLC7A11^high^ tumor cells.^2^ However, research on disulfidptosis remains in its infancy. The study presents for the first time a comprehensive investigation on disulfidptosis in BRCA. Diversity and complexity somatic mutations and copy number variations of disulfidptosis genes and their aberrant expression were found in breast tumors. Three disulfidptosis-based consensus clusters were established, with heterogeneous clinical, molecular, and immunogenomic features. A disulfidptosis-relevant signature (FHOD1, IL1R1, SPRY4, DOK4, TNN, ZMAT3, FEZ1, EMP1, WLS, ENPEP, RGS3, CCDC92, C11orf95, PTTG1IP, and SDC1) was proposed for reliably improving prognosis estimation. Low-risk patients more possibly responded to immune-checkpoint blockade, while high-risk patients were more sensitive to docetaxel. Disulfidptosis-relevant genes were proven to present abnormal expression in breast tumors. Disulfidptosis-relevant PTTG1IP overexpression led to aggressiveness and actin cytoskeleton formation in BRCA cells. Additionally, its overexpression induced tumor growth. These new findings proposed innovative disulfidptosis-based classification and signature for reflecting the heterogeneity of disulfidptosis in breast tumors, which might assist clinical-decision making.

To clarify this, we retrieved fourteen disulfidptosis genes and integrated genomic information of BRCA to comprehensively explore disulfidptosis expression patterns. The detailed baselines of the four enrolled cohorts are outlined in Table S1. Figure S1A illustrates the genomic position of experimentally identified disulfidptosis genes. Their expression was differential in breast tumors versus normal tissues (Fig. S1B), indicative of aberrant disulfidptosis in BRCA. Copy number variations frequently occurred in most disulfidptosis genes, notably CAPZB and FLNB (Fig. S1C). In addition, somatic mutations of disulfidptosis genes were frequent, especially MYH9 (26.9%), TLN1 (20.2%), FLNA (19.2%), and FLNB (19.2%) (Fig. S1D). The genetic alterations possibly affected the aberrant expression of most disulfidptosis genes. Both at the transcription and protein levels, disulfidptosis genes presented remarkable interactions (Fig. S1E, F). As expected, disulfidptosis genes primarily participated in modulating actin cytoskeleton (Figs. S1G–J), further proving their significance in disulfidptosis.

Subsequently, consensus clustering analysis was conducted based on disulfidptosis gene expression. CDF curves, tracking plot and consensus heatmap all demonstrated that the appropriate number of clusters was 3 (Figs. S2A–C; Fig. 1A). Thus, three consensus clusters were determined in BRCA, named as C1, C2, and C3, respectively. Principal component analysis proved the accuracy in disulfidptosis-based classification (Fig. 1B). Most disulfidptosis genes presented the most expression in the C1, followed by the C2, and lowest in the C3 (Fig. 1C). Thus, the C1, C2, and C3 clusters were regarded as high, moderate, and low disulfidptosis tumors. In addition, nearest template prediction (NTP) algorithm was applied to verify the stability and robustness of disulfidptosis consensus clusters. The result revealed that the three clusters displayed high reproducibility in the GSE17705 (Fig. S3A) and GSE58812 (Fig. S3C) datasets. Survival analysis results demonstrated a significant difference of clusters in GSE17705 (Fig. S3B) and GSE58812 (Fig. S3D). Cancer hallmark pathways exhibited heterogeneous activity in the three clusters, most of which exhibited stronger activity in the C1 (Fig. S4A). Moreover, heterogeneous clinical traits were detected among clusters, especially age (Fig. S4B). The C2 displayed the poorest OS, followed by the C1, and best in the C3 (Fig. 1D). We further evaluated the tumor heterogeneity among three cluster. In comparison to the C1, higher aneuploidy score, and fraction altered were found in the C2. Also, the C3 exhibited higher cancer-testis antigen (CTA) score, and homologous recombination defects versus the other two clusters. Number of segments was notably lower in the C1 than the other two clusters (Figs. S4C–G). Well-known immune checkpoints, and HLA molecules were also measured. Most had the lowest expression in the C2 versus the others (Fig. S5A, B). The immune microenvironment exhibited heterogeneity in the three clusters. There was the poorest abundance of most cell populations in the C2 (Fig. 1E). In addition, the C2 presented the lowest immune/stromal scores and the highest tumor purity (Figs. S5C–E). Altogether, the C2 was considered as immune-cold tumors with immune-escape mechanisms.

**Figure 1:**
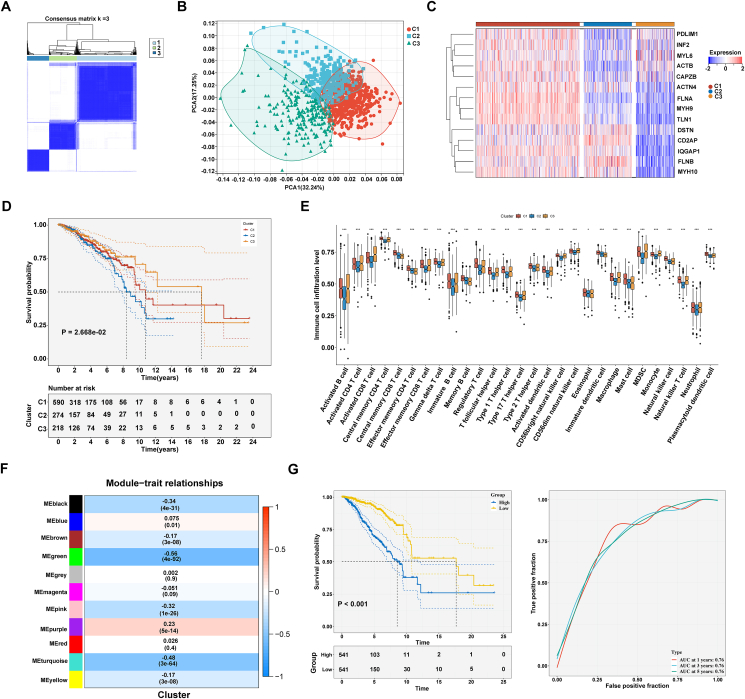
Construction of three disulfidptosis-based consensus clusters and risk model in BARC. **(A)** Consensus matrix at *k* = 3 based upon disulfidptosis genes. White to blue reflects consensus values ranging from 0 (never clustered together) to 1 (always clustered together). **(B)** Principal component analysis for proving the disulfidptosis-based consensus clusters marked by unique colors. **(C)** Expression of disulfidptosis genes across three clusters. Blue to red indicates down-to up-regulated expression. **(D)** Survival probability of three clusters. **(E)** Abundance of tumor-infiltrating immune cells across clusters. **(F)** Pearson analysis on co-expression modules with disulfidptosis-based classification. Color shade is proportional to association. **(G)** Survival probability of low- and high-risk groups, and receiver operator characteristic curves at 1, 3, and 5 years in TCGA-BRCA dataset.

Next, we tend to explore the genes associated with disulfidptosis expression pattern using weighted gene co-expression network analysis. To generate a scale-free co-expression network, the soft threshold power was 11 when scale-free R^2^ = 0.9 (Fig. S5F). Subsequently, through merging genes with similar expression patterns, eleven co-expression modules were generated, with the green module showing the strongest association (*r* = −0.56, *P* = 4e-92) with disulfidptosis consensus clusters (Fig. S5G, H; Fig. 1F). In this module, correlation coefficient of module membership with gene significance was up to 0.75, demonstrating the quality of disulfidptosis-relevant module establishment was excellent (Fig. S5I). Genes in the green module were regarded as disulfidptosis-relevant genes. Function enrichment analysis revealed that disulfidptosis-relevant genes were remarkably connected to regulation of actin cytoskeleton, and extracellular structure and matrix organization, further proving their relevance to disulfidptosis (Figs. S6A–D). In addition, they were linked with multiple tumorigenic pathways (*e.g.*, PI3K-Akt, MAPK, TGF-beta, and Wnt), indicative of their implications in BRCA onset and progression. Among disulfidptosis-relevant genes, 25 presented significant associations with patient survival (Table S2), which were subsequently utilized for LASSO analysis. Following the minimum lambda = 0.0088, 15 genes with coefficient ≠ 0 were eventually selected (Fig. S6E). A disulfidptosis-relevant signature was then generated using the risk formula mentioned in methods and materials. Then, TCGA-BRCA samples were classified into low- and high-risk subgroups based on the median risk score (Fig. S6F), with high-risk cases displaying poorer overall survival outcomes (Fig. 1G). The disulfidptosis-relevant signature demonstrated efficient prognosis estimation, as indicated by area under the curve values of 0.76 at one, three, and five years (Fig. 1G). The disulfidptosis-relevant signature was also validated in three independent cohorts: GSE17705 (Fig. S7A, B), GSE58812 (Fig. S7C, D), and METABRIC (Fig. S7E, F). Altogether, the disulfidptosis-relevant signature was reliable in estimating survival outcomes. In addition, combining uni- and multivariate-Cox regression methods, the disulfidptosis-relevant signature, age, and N stage were independently linked with patient survival (Fig. S8A, B). Based upon these variables, a nomogram was built for estimation of prognostic outcomes (Fig. S8C). Calibration curves proved that the nomogram accurately estimated survival outcomes (Fig. S8D). Additionally, decision curve analysis revealed the best net benefit from the nomogram (Figs. S8E–G). We further assess the association of disulfidptosis-relevant signature with molecular mechanisms, and therapeutic response. Tumorigenic pathways (cell cycle, ECM receptor interaction, PPAR and TGF-beta signaling pathways, *etc*.) presented the remarkable enrichment in high-risk tumors (Fig. S9A). In addition, high-risk tumors owned higher abundance of most immune cells (Fig. S9B). Overall, higher immune/stromal scores and lower tumor purity were found in high-risk tumors (Figs. S9C–E). Moreover, expression of most immune checkpoints was higher in high-risk group (Fig. S9F). TIDE algorithm estimated that low-risk group had more responders to immune checkpoint blockade (Figs. S9G–J). It was also found that high-risk patients presented stronger sensitivity to docetaxel (Fig. S9K).

We further experimentally verified expression of the disulfidptosis-relevant genes from the signature. FHOD1, IL1R1, SPRY4, ZMAT3, ENPEP, RGS3, PTTG1IP, and SDC1 were proven to present significant overexpression in MCF-7, SK-BR-3, and MDA-MB-231 cells versus MCF-10A cells (Fig. S10A–O). Also, DOK4, TNN, FEZ1, EMP1, WLS, CCDC92, and C11orf95 were proven to significantly down-regulate in these breast carcinoma cells than MCF-10A cells (Fig. S10A–O). Among the disulfidptosis-relevant genes, we further focused on PTTG1IP due to its undefined role in breast tumors. PTTG1IP expression was remarkably silenced by its specific siRNAs in MCF-7 and MDA-MB-231 cells (Fig. S11A, B). Additionally, its overexpression was achieved by transfection of PTTG1IP-overexpressed vectors (Fig. S11C, D). Migration ability was subsequently evaluated. Consequently, PTTG1IP knockdown notably alleviated migration of breast carcinoma cells, with antisense findings in the context of PTTG1IP-overexpressed vectors (Figs. S11E–H). Based upon phalloidin-marked F-actin, we found that silencing PTTG1IP remarkably attenuated F-actin formation of breast tumor cells. Oppositely, its overexpression improved F-actin formation (Figs. S12A–D). Altogether, overexpressed PTTG1IP results in aggressiveness and actin cytoskeleton formation in BRCA cells. Subsequently, the study observed the significance of PTTG1IP on tumor growth. It was found that proliferation of MCF-7 and MDA-MB-231 cells was alleviated by PTTG1IP knockdown (Figs. S13A–D). On the contrary, overexpressed PTTG1IP heightened cancer cell proliferation. In addition, apoptotic levels of breast tumor cells were reinforced by knockout PTTG1IP, with opposite consequences in the context of overexpressed PTTG1IP (Figs. S14A–D). Thus, PTTG1IP overexpression contributes to tumor overgrowth.

Collectively, we propose the susceptibility and expression patterns of disulfidptosis in breast tumors and suggest PTTG1IP as an actionable treatment target.

## Author contributions

KF conceived and designed the study. ZX and SJ conducted most of the experiments and data analysis, and wrote the manuscript. ML and CY participated in collecting data and helped draft the manuscript. All authors contributed to the article and approved the submitted version.

## Conflict of interests

The authors declare that the research was conducted in the absence of any commercial or financial relationships that could be construed as a potential conflict of interest.

## Funding

The research was supported by the Ningxia Reproductive Disease Clinical Medical Research Center Project (No. 2023LCYX003), Ningxia Hui Autonomous Region Natural Science Foundation Project (2022AAC03748), Ningxia Hui Autonomous Region Natural Science Foundation Project (No. 2021AAC03523), Basic research project of Yinchuan Maternal and Child Health Hospital (No. 2022NYFYCX05), Yinchuan Science and Technology Innovation Project (No. 2023SF25), and Basic research project of Yinchuan Maternal and Child Health Hospital (No. 2023NYFYCX01) (all China).

## Data availability

The original contributions presented in the study are included in the article or supplementary material. Further inquiries can be directed to the corresponding author.

## Figures and Tables

**figs1:**
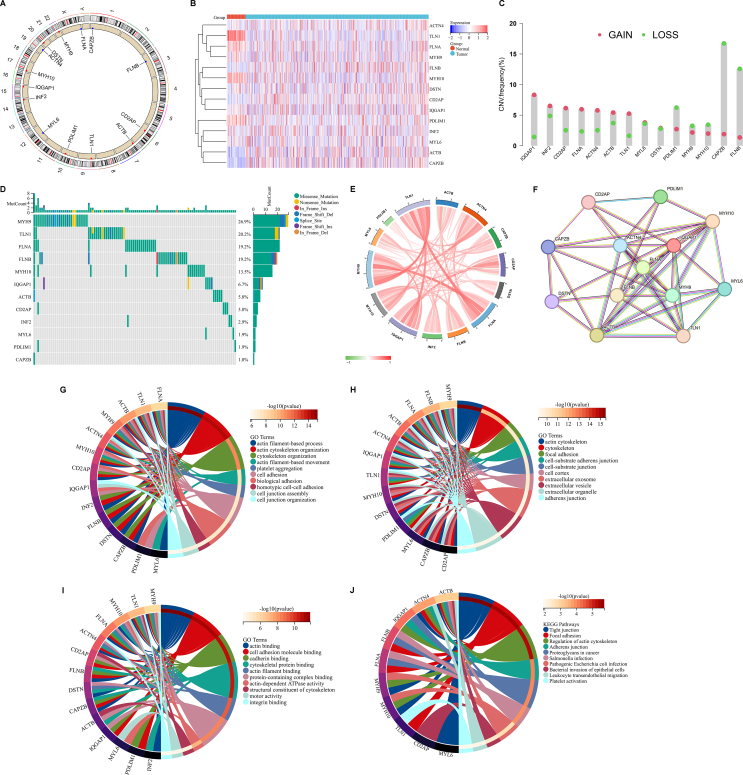


**figs2:**
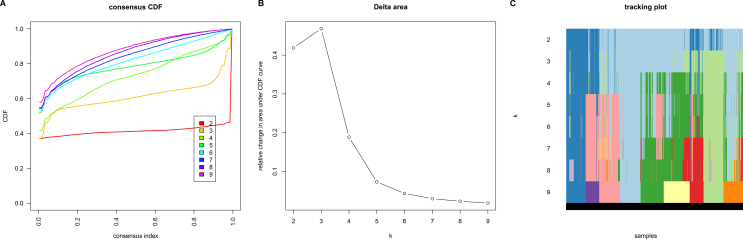


**figs3:**
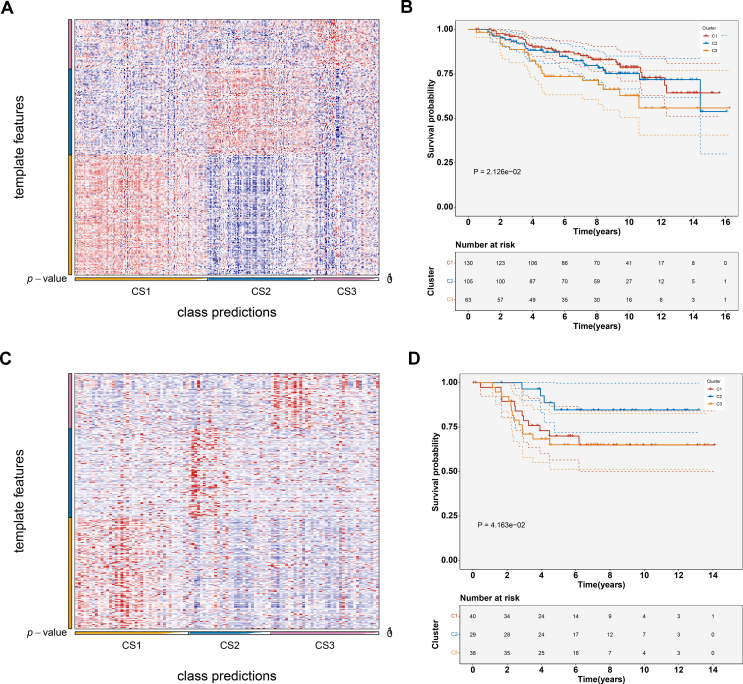


**figs4:**
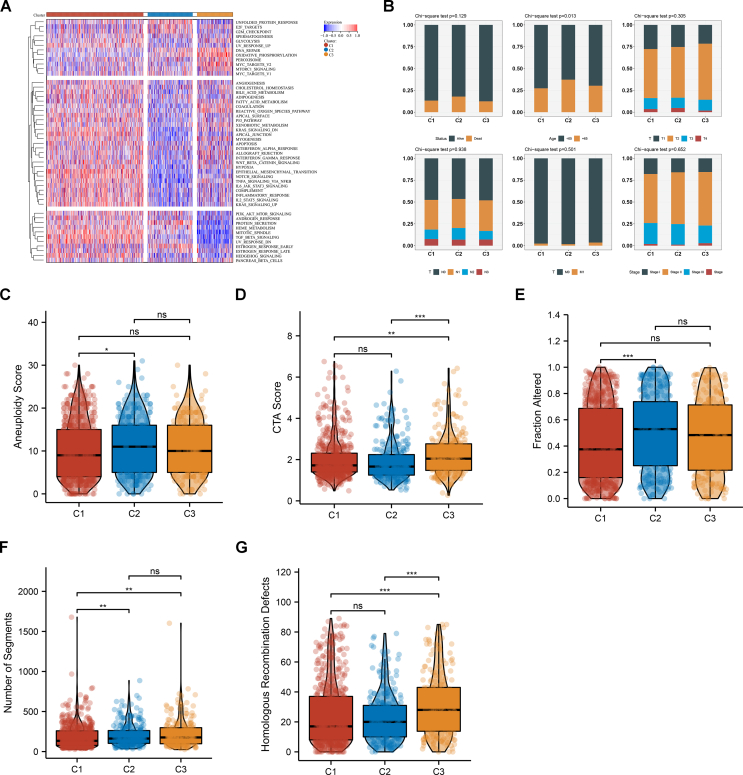


**figs5:**
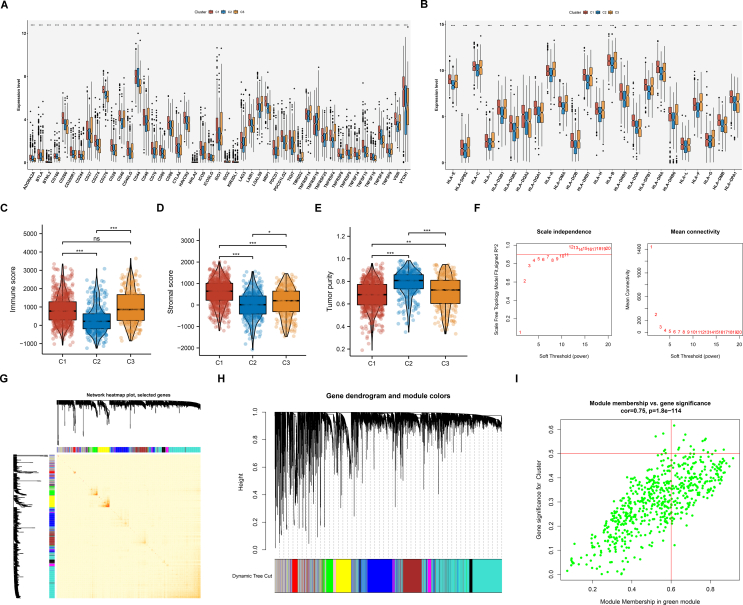


**figs6:**
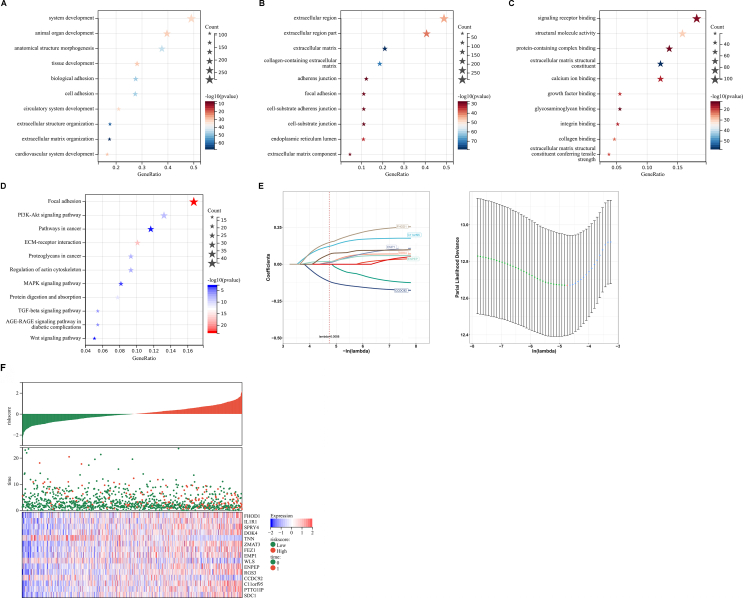


**figs7:**
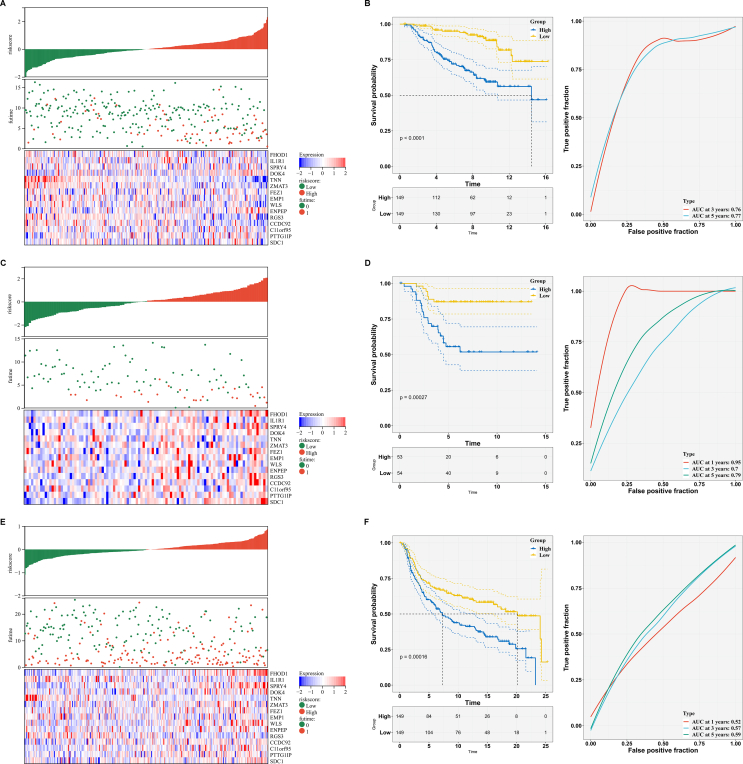


**figs8:**
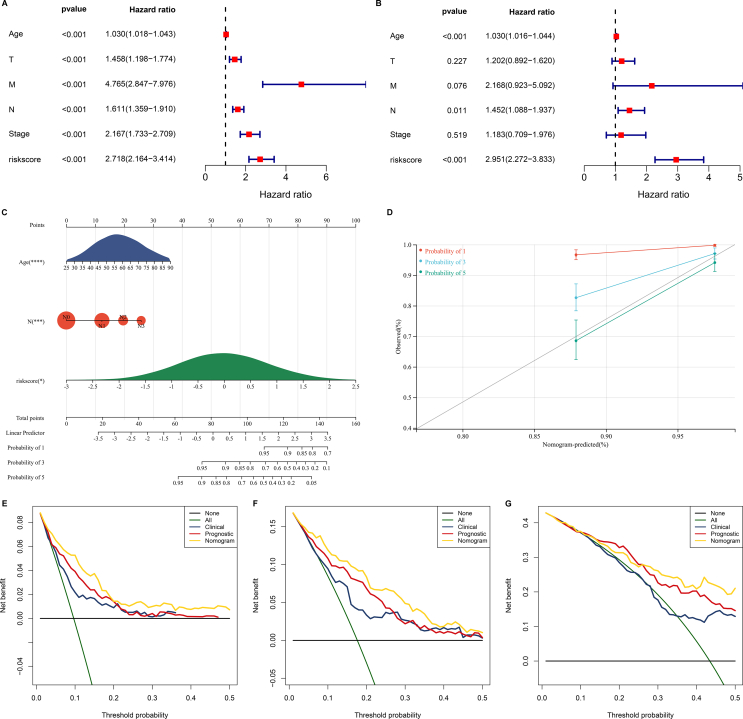


**figs9:**
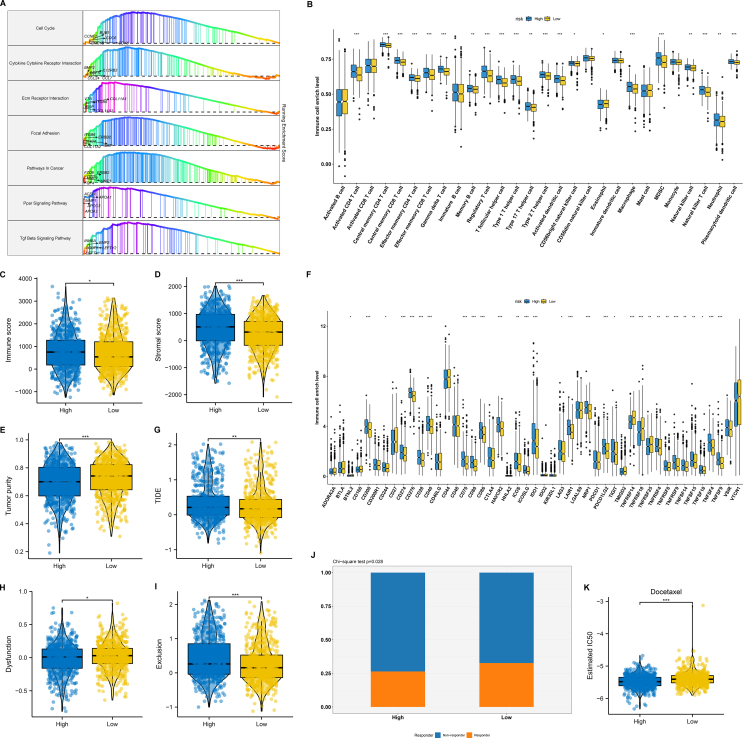


**figs10:**
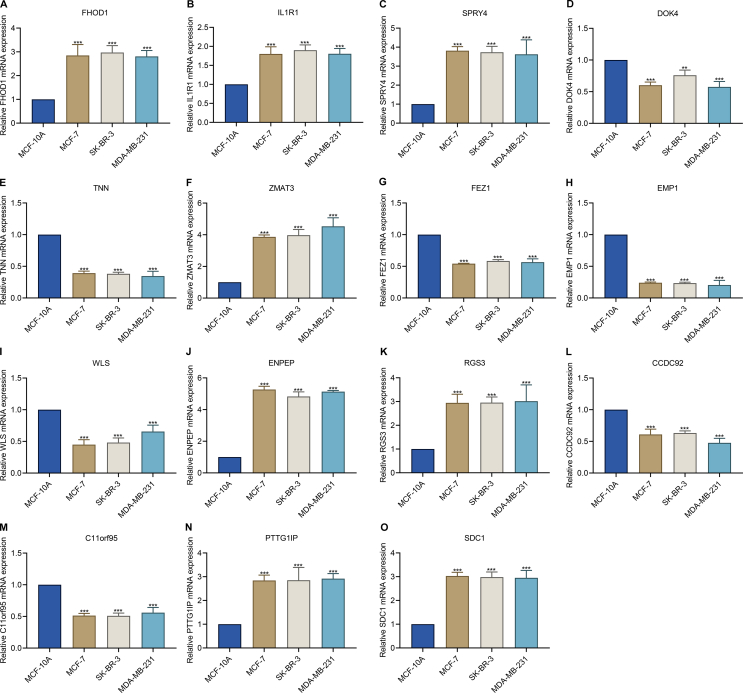


**figs11:**
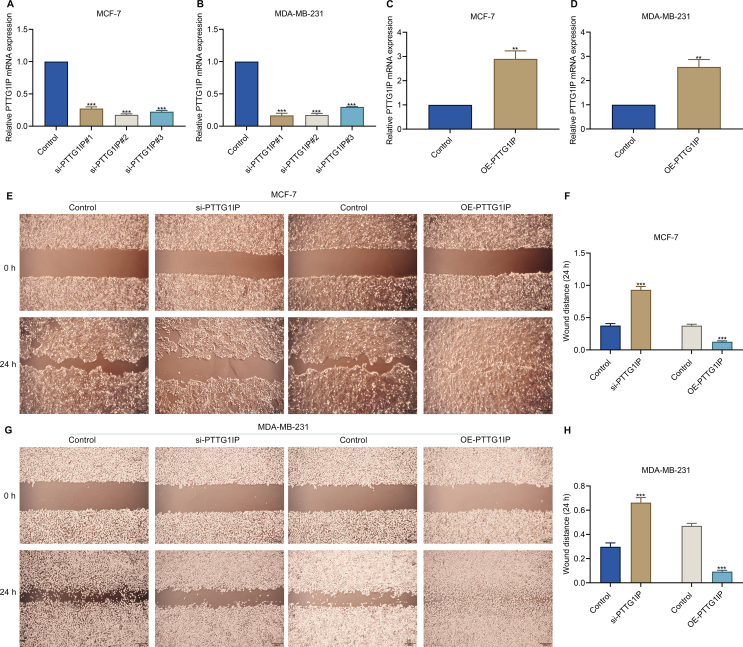


**figs12:**
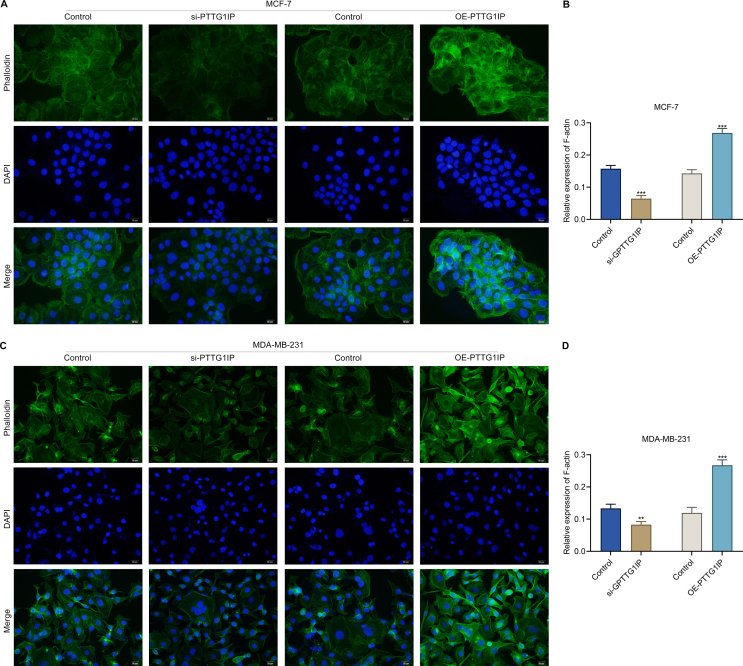


**figs13:**
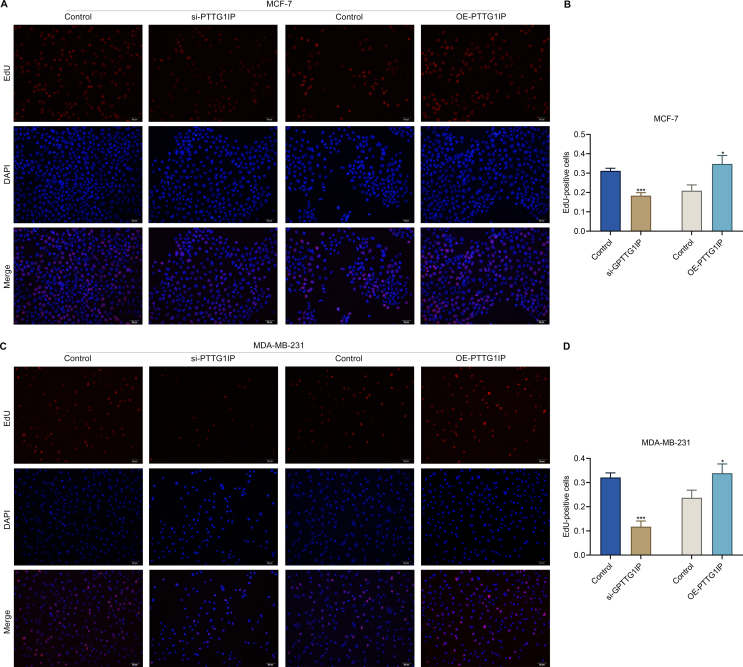


**figs14:**