# Supplementary material for: Disulfidptosis heterogeneity in breast cancer uncovers PTTG1IP as an actionable therapeutic target
Source: Genes Dis. 2024 Mar 5;12(1):101257. doi: 10.1016/j.gendis.2024.101257 (PMC12053626; doi:10.1016/j.gendis.2024.101257)
Supplement: Multimedia component 1 [file mmc1.docx]

**Materials and methods:**

**Data collection and curation**

Disulfidptosis genes were gathered from the previously published literature ^1^, encompassing ACTN4, ACTB, CD2AP, CAPZB, DSTN, FLNA, FLNB, INF2, IQGAP1, MYH10, MYL6, MYH9, PDLIM1, and TLN1. Their genomic information was visualized via RCircos package ^2^. They were inputted into the STRING online database (http://string-db.org/) ^3^ to unveil protein-protein interactions. RNA-seq profiles of 1109 breast tumors and 113 mammary specimens were acquired from The Cancer Genome Atlas Breast Carcinoma (TCGA-BRCA) (https://portal.gdc.cancer.gov/repository). Additionally, 1082 patients with complete clinical annotations were curated. Raw read counts from the cohort were converted to transcripts per kilobase million, with subsequent log-2 transformation. From the Gene Expression Omnibus (https://www.ncbi.nlm.nih.gov/geo/), GSE17705 (n=298) ^4^, and GSE58812 (n=107) cohorts on the Affymetrix platform were retrieved ^5^. Raw data from the Affymetrix were preprocessed through RMA approach of Affy package ^6^. The METABRIC cohort with primary invasive breast cancer (n=298) was gathered from the cbioportal for cancer genome (https://www.cbioportal.org/). The detailed baselines of the four enrolled cohorts are outlined in **Supplementary table 1**. The TCGA-BRCA cohort was set as training set, with the GSE17705, GSE58812, and METABRIC cohorts as external validation sets.

**Analysis of genetic alterations**

Copy number variations (CNVs) from the UCSC Xena data portal (http://xena.ucsc.edu/) were analyzed by GISTIC2.0 approach ^7^. Somatic mutations were also gathered and detected by use of maftools package ^8^.

**Functional annotation analysis**

ClusterProfiler package was employed for Gene Ontology (GO) and Kyoto Encyclopedia of Genes and Genomes (KEGG) enrichment analyses ^9^. Hallmark and “c2.cp.kegg.symbols” gene sets were acquired from the Molecular Signatures Database ^10^. Based upon them, enrichment score of hallmark pathways was estimated via GSVA package ^11^. In addition, gene set enrichment analysis (GSEA) ^12^ was conducted for KEGG pathways.

**Consensus clustering**

In accordance with the transcriptome profiling of disulfidptosis genes, consensus clustering was adopted for cluster discovery. The analysis was conducted through ConsensusClusterPlus ^13^. Each iteration took 80% of the samples for subsampling, and clustered each subsample into different k clusters (maximum k=9) via k-means algorithm in accordance with Euclidean distance. This process was repeated 50 times. Next, cumulative distribution function (CDF) curve, tracking plot, and consensus heatmap were adopted for choosing the optimal number of clusters. The clustering reliability was proven through principal component analysis (PCA).

**Tumor microenvironment analysis**

Immune cell infiltration was inferred via single-sample GSEA (ssGSEA) method based upon corresponding expression matrix of specific gene symbols ^14^. Immune / stromal scores, and tumor purity were analyzed utilizing ESTIMATE approach ^15^.

**Therapeutic response prediction**

Immune checkpoints, human leukocyte antigen (HLA) molecules, and Tumor Immune Dysfunction and Exclusion (TIDE) score were employed for reflecting immune checkpoint blockade (ICB) response. Utilizing pRRophetic package ^16^, IC50 value of chemotherapy drugs was computed based upon the Genomics of Drug Sensitivity in Cancer cell line expression spectrum ^17^.

**Weighted correlation network analysis (WGCNA)**

WGCNA package was adopted for generating a co-expression network ^18^. A suitable soft threshold power was chosen for satisfying a scale-free network. Conversion of a weighted adjacency matrix to a topological overlap matrix (TOM) was conducted. Subsequently, a corresponding dissimilarity (1-TOM) was built. Co-expression modules were merged through dynamic tree cutting method. Pearson correlation analysis was conducted on modules with disulfidptosis classification. A module with the highest correlation coefficient was regarded as classification-relevant module. Module membership and gene significance were computed, respectively. Genes in such module with module membership>0.6 & gene significance>0.5 were defined as disulfidptosis classification-relevant genes.

**Least absolute shrinkage and selection operator (LASSO)**

Univariate-cox regression method was employed for selecting prognostic disulfidptosis classification-relevant genes (p<0.05). Subsequently, LASSO analysis was executed via glmnet package ^19^, with removal of redundant genes to avoid overfitting under a minimum penalty coefficient.Risk score was computed for each case using with the formula: risk score = 0.204*FHOD1 + 0.078*IL1R1 + 0.03*SPRY4 + 0.053*DOK4 + (-0.411)*TNN + 0.344*ZMAT3 + 0.14*FEZ1 + 0.038*EMP1 + (-0.358)*WLS + 0.017*ENPEP + 0.135*RGS3 + (-0.192)*CCDC92 + 0.166*C11orf95 + 0.05*PTTG1IP + 0.1*SDC1. With the median risk score, cases were stratified into diverse risk groups. Overall survival (OS) difference was compared between groups, and receiver operating characteristic (ROC) curves were drawn for examining the prediction efficacy.

**Nomogram establishment**

Independent variables derived from uni- combined with multivariate-cox regression analyses were adopted to generate a nomogram. This process was achieved via rms package. The total score was added by each point corresponding to each variable in the nomogram for estimating patient survival. Calibration curves plotted for evaluating the prediction accuracy via bootstrap package. Moreover, decision curve analysis was conducted through rmda package for appraising the precision.

**Cell culture**

Human mammary epithelial cells (MCF-10A) were maintained in CM-0525 medium (Procell). Human breast carcinoma cells (MCF-7, SK-BR-3, and MDA-MB-231) were grown in Dulbecco's modified Eagle's medium (Procell). All media were added by 10% fetal bovine serum (FBS) as well as 1% penicillin-streptomycin (Invitrogen). All cells were cultivated in an incubator with 5% CO_2_ at 37 °C.

**Reverse transcription quantitative PCR (qPCR)**

RNA extraction was implemented utilizing RNAiso Plus reagent (Takara) in accordance with the manufacturer’s instructions, followed by cDNA synthesis via HiScript III RT SuperMix reagent (Vazyme). qPCR was conducted in CFX-96 Real time PCR Detection System (Bio-Rad) with SsoFast EvaGreen supermix (Bio-Rad). Relative expression was quantified with 2^-ΔΔCt^ approach. The primers for qPCR are summarized in **Table 1**.

**Table 1. Primer sequences for qPCR.**

| Gene name | Primer sequence |
| --- | --- |
| FHOD1 | 5’-CAAGTTGGAGGATTGTGCTCT-3’ (sense)  5’-GGTCCGAAGGATCAGCGTG-3’ (antisense) |
| IL1R1 | 5’-ATGAAATTGATGTTCGTCCCTGT-3’ (sense)  5’-ACCACGCAATAGTAATGTCCTG-3’ (antisense) |
| SPRY4 | 5’-TCTGACCAACGGCTCTTAGAC-3’ (sense)  5’-GTGCCATAGTTGACCAGAGTC-3’ (antisense) |
| DOK4 | 5’-ATGGCGACCAATTTCAGTGAC-3’ (sense)  5’-CTCCGGTAGATCCCGAGCTT-3’ (antisense) |
| TNN | 5’-GGAGATGTTCCGCTTCCCTAT-3’ (sense)  5’-GTCAGCGTCAACCTGAACCA-3’ (antisense) |
| ZMAT3 | 5’-AGAAGCCTTTTGGGCAGGAG-3’ (sense)  5’-TGCTGCATAGTAATTTCGGAGTT-3’ (antisense) |
| FEZ1 | 5’-CCACTGGTGAGTCTGGATGAA-3’ (sense)  5’-CGGAAGAAAAATTCTCAAGCTCG-3’ (antisense) |
| EMP1 | 5’-GTGCTGGCTGTGCATTCTTG-3’ (sense)  5’-CCGTGGTGATACTGCGTTCC-3’ (antisense) |
| WLS | 5’-TCCCTGGCTTACCGTGATGA-3’ (sense)  5’-GCATTTGAGTTTCCGTGGTACT-3’ (antisense) |
| ENPEP | 5’-AGAGGGCTCTAAGAGATACTGC-3’ (sense)  5’-CCACGGCAAGTCCCACTATT-3’ (antisense) |
| RGS3 | 5’-CCTCCTGGTCCCTCCCATTT-3’ (sense)  5’-ATGCCTCGGAGCATCTCTGTA-3’ (antisense) |
| CCDC92 | 5’-GAGTTACGATGAAGGTCCTCTGG-3’ (sense)  5’-TGTCTGTTCCGAACTTTTGACTG-3’ (antisense) |
| C11orf95 | 5’-CTCAAGGTGAGCACCATCAAG-3’ (sense)  5’-GCTCCTCAGGCGTGAAGTC-3’ (antisense) |
| PTTG1IP | 5’-GTCTGGACTACCCAGTTACAAGC-3’ (sense)  5’-CGCCTCAAAGTTCACCCAA-3’ (antisense) |
| SDC1 | 5’-CTGCCGCAAATTGTGGCTAC-3’ (sense)  5’-TGAGCCGGAGAAGTTGTCAGA-3’ (antisense) |
| GAPDH | 5’-ACAACTTTGGTATCGTGGAAGG-3’ (sense)  5’-GCCATCACGCCACAGTTTC-3’ (antisense) |

**Cell transfection**

When the cellular density reached 60%~70%, jetPRIME® transfection reagent (Polyplus) was employed for transfecting cells with small interfering RNAs (siRNAs) of PTTG1IP (si-PTTG1IP) (RiboBio) or PTTG1IP expression vector (human PTTG1IP full-length cDNA ORF Clone) (OE-PTTG1IP) as instructed by the manufacturer.

**EdU staining**

EdU staining assay was conducted based upon the manufacturer's instructions (Beyotime). Culture medium (100 µL/well) plus 50 µmol/L EdU was added to the plate and cultivated at 37 °C for 3 h for labeling the DNA. Next, cells were fixed by 4% paraformaldehyde as well as permeabilized by 0.5% PBS-Triton X-100. Prepared reaction mixture plus fluorescent dye was added, and the plate was cultivated at 25 °C away from the light. The nuclei were subsequently dyed by DAPI for half one hour. Cells were imaged utilizing a fluorescence microscope (ZEISS).

**TUNEL staining**

TUNEL staining assay was implemented as instructed by the manufacturer. Cells were fixed by 4% paraformaldehyde lasting half one hour. Subsequently, the sections were dyed utilizing TUNEL Apoptosis Assay Kit (Beyotime). The sections were photographed under a fluorescence microscope (ZEISS).

**Wound healing assay**

2 × 10^5^ cells were inoculated in 6-well plates. When the cellular density covered the entire well, serum-free media were adopted for culturing the cells for 6 h. Scratches were made via a 200-μL pipette tip, with subsequent removal of cellular debris by PBS. Images of scratches were acquired under a microscope (ZEISS) at 0, and 24 h.

**Immunofluorescence**

Cells were grown on glass cover slips in a 24 well plate. The next day, after removing cell medium, cell fixation by 4% paraformaldehyde and permeation by 0.5% Triton X-100/PBS were conducted, respectively. After blockade by 1% BSA/PBS, cells were probed with Alexa Fluor 488-conjugated phalloidin (Thermo Fisher). Images were photographed utilizing a fluorescence microscope (ZEISS).

**Statistical analysis**

All data are expressed as the mean ± standard deviation, and were analyzed through R software (version 4.0.3) or GraphPad Prism (version 9.0.1). Statistical analysis of 2 groups was evaluated via Student’s t or Wilcoxon test. One-way ANOVA was adopted for comparing ≥3 groups. Through Chi-square test, categorical variables were compared. Correlation analysis was achieved by Pearson test. Statistical significance was set as p<0.05.

1. Liu, X.; Nie, L.; Zhang, Y.; Yan, Y.; Wang, C.; Colic, M.; Olszewski, K.; Horbath, A.; Chen, X.; Lei, G.; Mao, C.; Wu, S.; Zhuang, L.; Poyurovsky, M. V.; James You, M.; Hart, T.; Billadeau, D. D.; Chen, J.; Gan, B., Actin cytoskeleton vulnerability to disulfide stress mediates disulfidptosis. *Nat Cell Biol* **2023,** *25* (3), 404-414.

2. Zhang, H.; Meltzer, P.; Davis, S., RCircos: an R package for Circos 2D track plots. *BMC Bioinformatics* **2013,** *14*, 244.

3. Szklarczyk, D.; Morris, J. H.; Cook, H.; Kuhn, M.; Wyder, S.; Simonovic, M.; Santos, A.; Doncheva, N. T.; Roth, A.; Bork, P.; Jensen, L. J.; von Mering, C., The STRING database in 2017: quality-controlled protein-protein association networks, made broadly accessible. *Nucleic Acids Res* **2017,** *45* (D1), D362-d368.

4. Symmans, W. F.; Hatzis, C.; Sotiriou, C.; Andre, F.; Peintinger, F.; Regitnig, P.; Daxenbichler, G.; Desmedt, C.; Domont, J.; Marth, C.; Delaloge, S.; Bauernhofer, T.; Valero, V.; Booser, D. J.; Hortobagyi, G. N.; Pusztai, L., Genomic index of sensitivity to endocrine therapy for breast cancer. *J Clin Oncol* **2010,** *28* (27), 4111-9.

5. Jézéquel, P.; Loussouarn, D.; Guérin-Charbonnel, C.; Campion, L.; Vanier, A.; Gouraud, W.; Lasla, H.; Guette, C.; Valo, I.; Verrièle, V.; Campone, M., Gene-expression molecular subtyping of triple-negative breast cancer tumours: importance of immune response. *Breast Cancer Res* **2015,** *17*, 43.

6. Gautier, L.; Cope, L.; Bolstad, B. M.; Irizarry, R. A., affy--analysis of Affymetrix GeneChip data at the probe level. *Bioinformatics* **2004,** *20* (3), 307-15.

7. Mermel, C. H.; Schumacher, S. E.; Hill, B.; Meyerson, M. L.; Beroukhim, R.; Getz, G., GISTIC2.0 facilitates sensitive and confident localization of the targets of focal somatic copy-number alteration in human cancers. *Genome Biol* **2011,** *12* (4), R41.

8. Mayakonda, A.; Lin, D. C.; Assenov, Y.; Plass, C.; Koeffler, H. P., Maftools: efficient and comprehensive analysis of somatic variants in cancer. *Genome Res* **2018,** *28* (11), 1747-1756.

9. Yu, G.; Wang, L. G.; Han, Y.; He, Q. Y., clusterProfiler: an R package for comparing biological themes among gene clusters. *Omics* **2012,** *16* (5), 284-7.

10. Liberzon, A.; Birger, C.; Thorvaldsdóttir, H.; Ghandi, M.; Mesirov, J. P.; Tamayo, P., The Molecular Signatures Database (MSigDB) hallmark gene set collection. *Cell Syst* **2015,** *1* (6), 417-425.

11. Hänzelmann, S.; Castelo, R.; Guinney, J., GSVA: gene set variation analysis for microarray and RNA-seq data. *BMC Bioinformatics* **2013,** *14*, 7.

12. Subramanian, A.; Tamayo, P.; Mootha, V. K.; Mukherjee, S.; Ebert, B. L.; Gillette, M. A.; Paulovich, A.; Pomeroy, S. L.; Golub, T. R.; Lander, E. S.; Mesirov, J. P., Gene set enrichment analysis: a knowledge-based approach for interpreting genome-wide expression profiles. *Proc Natl Acad Sci U S A* **2005,** *102* (43), 15545-50.

13. Wilkerson, M. D.; Hayes, D. N., ConsensusClusterPlus: a class discovery tool with confidence assessments and item tracking. *Bioinformatics* **2010,** *26* (12), 1572-3.

14. Bindea, G.; Mlecnik, B.; Tosolini, M.; Kirilovsky, A.; Waldner, M.; Obenauf, A. C.; Angell, H.; Fredriksen, T.; Lafontaine, L.; Berger, A.; Bruneval, P.; Fridman, W. H.; Becker, C.; Pagès, F.; Speicher, M. R.; Trajanoski, Z.; Galon, J., Spatiotemporal dynamics of intratumoral immune cells reveal the immune landscape in human cancer. *Immunity* **2013,** *39* (4), 782-95.

15. Yoshihara, K.; Shahmoradgoli, M.; Martínez, E.; Vegesna, R.; Kim, H.; Torres-Garcia, W.; Treviño, V.; Shen, H.; Laird, P. W.; Levine, D. A.; Carter, S. L.; Getz, G.; Stemke-Hale, K.; Mills, G. B.; Verhaak, R. G., Inferring tumour purity and stromal and immune cell admixture from expression data. *Nat Commun* **2013,** *4*, 2612.

16. Geeleher, P.; Cox, N.; Huang, R. S., pRRophetic: an R package for prediction of clinical chemotherapeutic response from tumor gene expression levels. *PLoS One* **2014,** *9* (9), e107468.

17. Yang, W.; Soares, J.; Greninger, P.; Edelman, E. J.; Lightfoot, H.; Forbes, S.; Bindal, N.; Beare, D.; Smith, J. A.; Thompson, I. R.; Ramaswamy, S.; Futreal, P. A.; Haber, D. A.; Stratton, M. R.; Benes, C.; McDermott, U.; Garnett, M. J., Genomics of Drug Sensitivity in Cancer (GDSC): a resource for therapeutic biomarker discovery in cancer cells. *Nucleic Acids Res* **2013,** *41* (Database issue), D955-61.

18. Langfelder, P.; Horvath, S., WGCNA: an R package for weighted correlation network analysis. *BMC Bioinformatics* **2008,** *9*, 559.

19. Wang, Z.; Yao, J.; Dong, T.; Niu, X., Definition of a Novel Cuproptosis-Relevant lncRNA Signature for Uncovering Distinct Survival, Genomic Alterations, and Treatment Implications in Lung Adenocarcinoma. *J Immunol Res* **2022,** *2022*, 2756611.

**Additional files:**

**Figure S1** Multi-omics analysis of disulfidptosis genes in breast tumors. **(A)** The Circos diagram illustrating the genomic location of disulfidptosis genes. The number in the outermost circle indicates the position of the chromosome, while gene name is localized inside the circle. **(B)** The heatmap visualizing the transcript values of disulfidptosis genes in normal and tumor tissues from TCGA-BRCA cohort. The color from blue to red indicates low to high expression. **(C)** Lollipop chart of the copy number variation frequency of each disulfidptosis gene. Red, copy number amplification; green, copy number deletion. **(D)** The waterfall plot depicting the somatic mutation frequency of disulfidptosis genes. The mutation forms are colored by unique colors. The mutation frequency is presented in the right panel. **(E)** The Circos chart displaying the mRNA correlation of any two disulfidptosis genes. The color from green to red denotes negative to positive correlation. **(F)** Protein-protein interactions of disulfidptosis genes. **(G–I)** The enrichment on biological processes, cellular components, and molecular functions by disulfidptosis genes. **(J)** The enriched KEGG results based upon disulfidptosis genes.

**Figure S2** Consensus clustering of TCGA-BRCA samples. **(A)** CDF curves at k = 2~9. **(B)** Relative change in area under CDF curves. **(C)** Tracking plot for diverse sample classifications. CDF, cumulative distribution function.

**Figure S3** The reproducibility of the disulfidptosis consensus clusters in multiple datasets was validated with the nearest template prediction (NTP) algorithm. **(A)** Transcriptome profiling of the template features across disulfidptosis consensus clusters in the GSE17705. **(B)** Kaplan Meier curves of overall survival (OS) among clusters in the GSE17705. **(C)** Transcriptome profiling of the template features across disulfidptosis consensus clusters in the GSE58812. **(D)** Kaplan Meier curves of overall survival (OS) among clusters in the GSE58812.

**Figure S4** Three disulfidptosis-based consensus clusters with diverse clinical and molecular features. **(A)** Activity of hallmark pathways in diverse clusters. Blue to red represents inactivation to activation. **(B)** Distribution of clinical parameters across clusters. **(F)** Survival probability of three clusters. **(C–G)** Comparison of aneuploidy score, CTA score, fraction altered, number of segments, and homologous recombination defects among the three clusters. ns, not significant; ^*^*P* < 0.05, ^**^*P* < 0.01, ^***^*P* < 0.001.

**Figure S5** Analysis of immunogenomic features in three disulfidptosis-based consensus clusters, and selection of disulfidptosis-relevant genes. **(A, B)** Expression of immune checkpoints and HLA molecules in the three clusters. **(C–E)** Immune and stromal scores and tumor purity among clusters. **(F)** Scale-free topology fit indexes and mean connectivity values under a gradient of soft-threshold powers. **(G)** The heatmap illustrating the correlation between genes. Yellow to red denotes weak to strong association. **(H)** Generation of co-expression modules marked by unique colors. **(I)** Relationships of module membership of green module with gene signature of disulfidptosis-based classification. ns, not significant; ^*^*P* < 0.05, ^**^*P* < 0.01, ^***^*P* < 0.001.

**Figure S6** Generation of a disulfidptosis-relevant signature for prognosis estimation. **(A–D)** Biological process, cellular component, molecular function, and KEGG pathway enrichment analyses on disulfidptosis-relevant genes. **(E)** LASSO coefficient profiling under a minimum lambda. **(F)** Distribution of risk score, survival time, and status, and expression of selected disulfidptosis-relevant genes.

**Figure S7** External validation of the effectiveness of disulfidptosis-relevant signature in estimating patient survival. **(A)** Risk score, survival time, and gene expression in the GSE17705. **(B)** Survival probability of low- and high-risk groups and one-, three- and five-year ROC curves in the GSE17705. **(C)** Risk score, survival time, and gene expression in the GSE58812. **(D)** Survival probability of two groups and one-, three- and five-year ROC curves in the GSE58812. **(E)** Risk score, survival duration, and gene expression in the METABRIC. **(F)** Survival probability of two groups and ROC curves at 1, 3, and 5 years in the METABRIC. ROC, receiver operator characteristic.

**Figure S8** Definition of a nomogram composed of disulfidptosis-relevant signature, age, and N stage. **(A, B)** Uni- and multivariate-Cox regression approaches on disulfidptosis-relevant signature and clinical parameters with patient survival. **(C)** Nomogram construction based upon independent prognostic variables. **(D)** The calibration curves demonstrating the accuracy of the nomogram-estimated prognosis. **(E–G)** The decision curve analysis evaluating the net benefit from the nomogram at the threshold of 1, 3, and 5 years.

**Figure S9** Associations of disulfidptosis-relevant signature with molecular mechanisms, and therapeutic response. **(A)** GSEA for the enriched KEGG pathways in high- versus low-risk groups. **(B)** Abundance of immune cells in high- and low-risk tumors. **(C–E)** Immune and stromal scores and tumor purity in two groups. **(F)** Expression of immune checkpoint molecules in two groups. **(G–J)** TIDE, dysfunction and exclusion scores, and estimated responders to immune checkpoint blockade. **(K)** IC50 of docetaxel estimated in two groups. ^*^*P* < 0.05, ^**^*P* < 0.01, ^***^*P* < 0.001.

**Figure S10** Experimental validation of expression of disulfidptosis-relevant genes from the signature. **(A–O)** Quantitative PCR for measuring (A) FHOD1, (B) IL1R1, (C) SPRY4, (D) DOK4, (E) TNN, (F) ZMAT3, (G) FEZ1, (H) EMP1, (I) WLS, (J) ENPEP, (K) RGS3, (L) CCDC92, (M) C11orf95, (N) PTTG1IP, and (O) SDC1 expression in MCF-10A, MCF-7, SK-BR-3, and MDA-MB-231 cells. ^**^*P* < 0.01, ^***^*P* < 0.001.

**Figure S11** PTTG1IP motivates aggressiveness of breast cancer cells. **(A, B)** PTTG1IP expression in MCF-7 and MDA-MB-231 cells with its specific siRNAs. **(C, D)** PTTG1IP expression in MCF-7 and MDA-MB-231 cells when transfected with PTTG1IP-overexpressed vectors. **(E–H)** Wound healing at 0 h and 24 h for MCF-7 and MDA-MB-231 cells under si-PTTG1IP- or PTTG1IP-overexpressed vectors. Bar scale, 200 μm. ^**^*P* < 0.01, ^***^*P* < 0.001.

**Figure S12** PTTG1IP facilitates actin cytoskeleton formation in breast cancer cells. **(A–D)** Phalloidin-marked F-actin in MCF-7 and MDA-MB-231 cells in the context of si-PTTG1IP- or PTTG1IP-overexpressed vectors. Bar scale, 20 μm. ^**^*P* < 0.01, ^***^*P* < 0.001.

**Figure S13** PTTG1IP improves proliferation of breast cancer cells. **(A–D)** EdU staining of MCF-7 and MDA-MB-231 cells in the context of si-PTTG1IP- or PTTG1IP-overexpressed vectors. Bar scale, 50 μm. ^*^*P* < 0.05, ^***^*P* < 0.001.

**Figure S14** PTTG1IP alleviates apoptosis of breast cancer cells. **(A–D)** TUNEL staining for MCF-7 and MDA-MB-231 cells in the context of si-PTTG1IP- or PTTG1IP-overexpressed vectors. Bar scale, 50 μm. ^**^*P* < 0.01, ^***^*P* < 0.001.

**Table S1** Clinical traits of breast cancer from the TCGA-BRCA, GSE17705, GSE58812, and METABRIC cohorts.

**Table S2** Prognostic disulfidptosis-relevant genes.
